# Editorial: Chrono-immunonutrition in aquaculture towards robust and resilient fish

**DOI:** 10.3389/fimmu.2024.1547738

**Published:** 2025-01-31

**Authors:** Byron Morales-Lange, Maria del Mar Ortega-Villaizan, Sérgio D. C. Rocha, Ruth Montero, Margareth Øverland

**Affiliations:** ^1^ Department of Animal and Aquacultural Sciences, Faculty of Biosciences, Norwegian University of Life Sciences, Ås, Norway; ^2^ Instituto de Investigación, Desarrollo e Innovación en Biotecnología Sanitaria de Elche, Universidad Miguel Hernández, Elche, Spain

**Keywords:** fish farming, immunonutrition, nutritional-programming, novel feeds, bioactive compounds, gut health, systemic immune responses

## Introduction

According to the Food and Agriculture Organization (FAO) from the United Nations, aquaculture fish production surpasses fisheries and it will continue to grow globally in the coming decades (1), contributing to high quality food production for the increasing human population. Thus, fish farming is an important actor to achieve UN-Sustainable Development Goals (SDGs) such as SDG 2 (Zero hunger), SDG 12 (Responsible consumption and production) and SDG 14 (Life below water) (2). However, this industry faces several challenges that negatively impact its sustainability. For instance, sub-optimal nutrition, infectious diseases (caused by parasites, bacteria and virus) and environmental problems (e.g., increased seawater temperature, algal blooms) can decrease fish performance and overall health, as well as increase fish mortality, leading to economic losses for the aquaculture sector (3–5). Therefore, to achieve a resilient fish capable of coping with stressful conditions and to strengthen aquaculture to meet high quality protein demands from an increasing global population, it is essential to deepen the understanding of fish metabolism, immune responses against pathogens and host-microbiota interaction.

In mammals, the “immunonutrition” concept has been described as nutritional interventions to produce health-related effects, such as reducing the risk of pathogen infection and improving healing process (6, 7). To contribute with knowledge on this topic in fish with economic importance (i.e., Atlantic salmon, *Salmo salar*; rainbow trout, *Oncorhynchus mykiss*; European seabass, *Dicentrarchus labrax*), the scientific articles in the Research Topic “*Chrono-Immunonutrition in Aquaculture towards Robust and Resilient fish*” focused on alternative feed ingredients, fish growth performance, immune modulation, health responses against challenges (e.g., exposure to microbe-associated molecular patterns and sub-optimal nutrition), as well as microbiota characterization and its interaction with the host.

**Figure 1:**
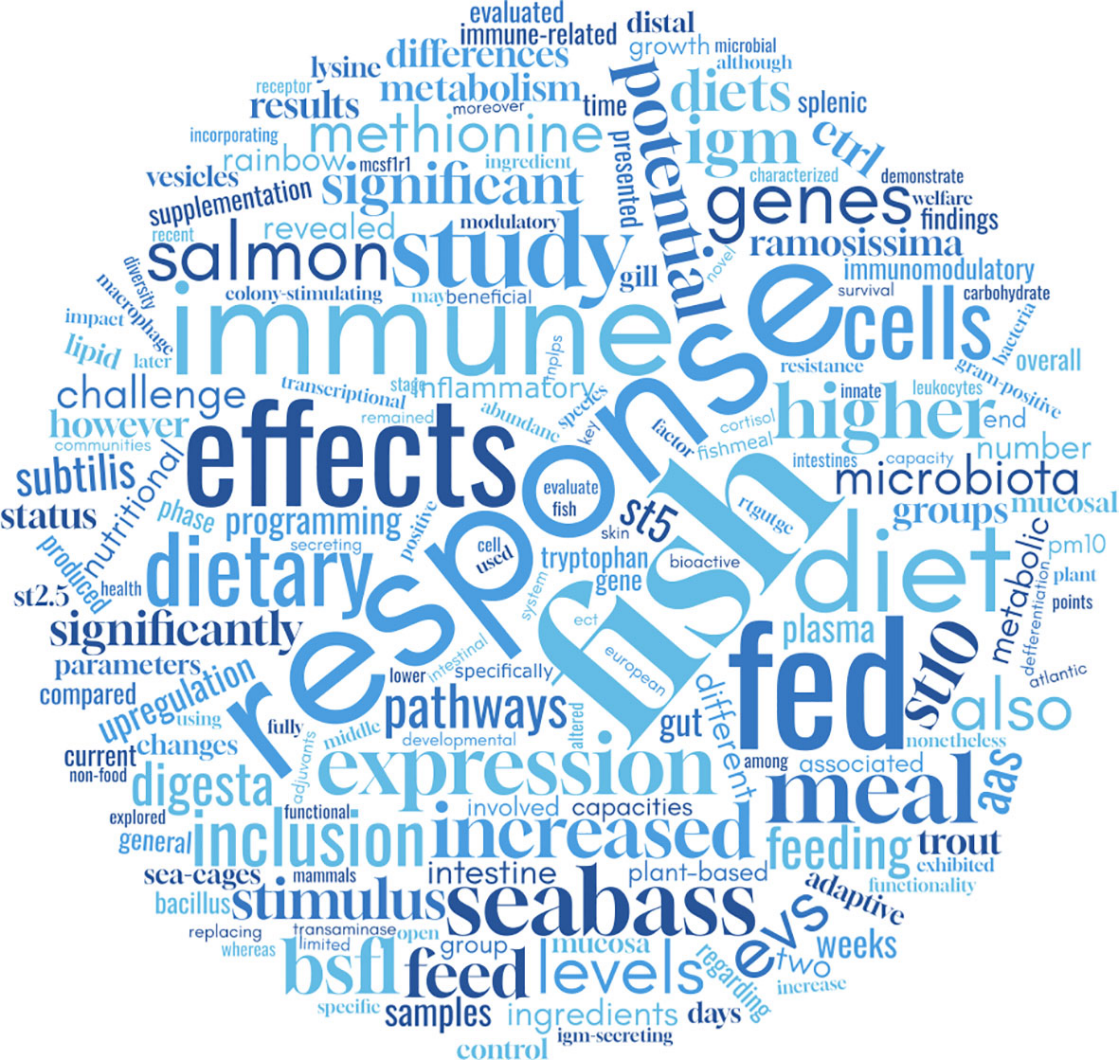
Word cloud based on abstract analysis related to the Research Topic “Chrono-Immunonutrition in Aquaculture towards Robust and Resilient fish”. Credits to Wordclouds.com.

Furthermore, an analysis based on the different abstracts comprising this Research Topic revealed that words such as “fish immune response”, “diet effects”, “feed”, “microbiota”, “gene expression”, “stimulus” and “meal” were the most used concepts by the different authors (**Figure 1**). This proposes the importance of immunonutrition as an experimental approach to modulate fish physiology and to overcome the needs of the aquaculture industry.

## Gut as a primary target for immunomodulation and the potential coordination of systemic responses

The gut is as a mucosa-associated lymphoid tissue (MALT) that contains cells (e.g., granulocytes, macrophages, antigen-presenting cells and lymphocytes) and molecular components such cytokines (e.g., polarizing, pro- and anti-inflammatory) and effector molecules (e.g., enzymes, antimicrobial peptides, immunoglobulins) (8, 9), which can coordinate innate and adaptive immune mechanisms locally in the distal intestine as well as at the systemic level (8, 9) linked to immune-related organs [e.g., head kidney (10), spleen (11), and liver (12, 13)]. Therefore, the gut is a primary target for nutritional strategies that seek to improve overall fish health and welfare, without compromising their growth performance.

Recently, Vicente-Gil et al. reported that extracellular vesicles (EVs) from *Bacillus subtilis* induced a differential gene expression on rainbow trout intestinal cells (RTgutGC). For instance, the authors detected an upregulation of pro-inflammatory cytokines (*il-1β, il-8*), antimicrobial peptides (*hepcidin*, *cathelicidin 2*) and biomarkers involved in intestinal barrier integrity and homeostasis (*claudin 3, ZO-1*), as well as mucin production (*imuc*). In addition, primary cultures of trout splenic leukocytes stimulated with EVs from *B. subtilis* showed a higher number of IgM-secreting cells. Also, splenic IgM^+^ B cells increased MHC II surface levels and antigen-processing capacities. Interestingly, antigen presentation process is the bridge between the innate and adaptive mechanisms of the immune response (14) and these *in vitro* results suggest the immunomodulatory potential of EVs from *B. subtilis* as adjuvants or immunostimulants for aquaculture.

Linked to nutrient absorption and utilization, Martin et al., investigated the immunomodulatory properties of L-methionine on spleen leukocytes from rainbow trout. Following this characterization, they evaluated the potential effects of dietary supplementation with this amino acid during a one-month experiment with rainbow trout, followed by anal immunization with 2,4,6-trinitrophenyl hapten conjugated to lipopolysaccharide (TNP-LPS). Their data showed that fish fed MET1 (diet containing 44% more L-methionine than commercial control diet) had a significantly higher number of blood IgM-secreting cells. Also, after the anal immunization, trout fed MET1 had higher titers of TNP-specific IgMs. This demonstrates the positive effects of methionine supplementation to modulate adaptive immune responses in rainbow trout, which could contribute to strengthening the effect of immunological strategies (i.e., vaccines) currently used in aquaculture to prevent and/or control infectious biological agents.

## Nutritional programming, novel feeds and aquaculture sustainability

To promote more sustainable aquaculture and increase animal welfare, nutritional programming can be part of the solution. Nutritional programming is influenced by the source of functional compounds (ingredients or additives), their feed inclusion level, feeding period, season, as well as the stage of animal development (15). In Atlantic salmon, Tawfik et al. reported that nutritional programming, using plant-based diets for two weeks at early life stages, can improve physiological responses when fish are exposed to a similar feeds 20 weeks later. For example, after a dietary challenge with plant ingredients, Atlantic salmon showed changes in their gut transcriptome, including regulatory epigenetic responses and lipid metabolism were up-regulated, while genes involved in innate immune response were down-regulated. These results are relevant, when considering the shift that aquaculture has made from marine to plant-based ingredients over the past 20 years. Atlantic salmon is a carnivorous fish and the use of plant ingredients has been limited due to the documented negative health effects associated to the presence of antinutrients (e.g., saponins, phytic acid, enzyme inhibitors and lectins), which can reduce growth performance and cause intestinal inflammation (16, 17).

In another important aquaculture species, European seabass fed two months with a novel feed containing 10% *Salicornia ramosissima* (a halotolerant succulent) showed no differences in fish growth, while a modulated immune gene expression in the head kidney (i.e., upregulation of *mcsf1r1* and *cd8β*) (Machado et al.) was detected. Then, after an intraperitoneal bacterial challenge with *Photobacterium damselae piscicida*, fish fed 10% *S. ramosissima* had a higher number of peritoneal leucocytes and an upregulation of *mcsf1r1*, *il-1β* and *gpx* (Machado et al.). This supports the idea that functional feeds containing bioactive ingredients or additives can modulate the immune response of fish to address challenges without compromising their performance. Regarding microbiota modulation (trough 16S rRNA gene sequencing), Monteiro et al. have proven that juvenile European seabass fed a diet with 40% fishmeal replaced by polychaete-based meal (from *Alitta virens*) had a lower relative abundance of *Mycobacterium*, *Taeseokella* and *Clostridium* both in mucosa and intestinal digesta samples. Then, using a predictive functional analysis of bacterial communities in the mucosa, the authors described differences in phenylalanine metabolism and sulfur relay system, whereas valine, leucine, thyroid hormone signaling, and isoleucine degradation and secretion system pathways varied in the digesta samples. This type of analysis is important since the gut is a key organ for host-microbiota interactions, which can be related to amino acid metabolism, secretion-related pathways and the production of secondary metabolites. Moreover, host-microbiota interactions can be part of the link between nutrition, immune response and host health (18, 19).

In industrial aquafeed production, incorporating novel ingredients derived from the bioconversion of underutilized biomass or industrial side-streams can help to reduce the environmental footprint of aquaculture (20). This is particularly important as feeds are the largest contributor to its carbon footprint in fish production (21). However, the inclusion of novel ingredients in fish feeds is still low (22) and their evaluation during field trials is limited. Nevertheless, Radhakrishnan et al. studied Atlantic salmon fed black soldier fly larvae (BSFL) meal at 5% and 10% for 13 months in open sea-cages, replicating real farm conditions. Their results showed that while 10% BSFL meal did significant effected general growth performance, welfare or survival, 5% BSFL meal induced positive responses in mucosal tissues, as well as hematological and gene expression profiles of salmon. For instance, an upregulation of *il-1β* in skin and gills, along with *mm-9* and *mucin18* in gills were detected, as well as a decreased cortisol response, higher mucus secretions, and an increased number of erythrocytes. These findings suggest that 5% inclusion of BSFL meal can modulate fish immune response under farm conditions. Thus, depending on regulatory restrictions, BSFL meal could be an alternative ingredient to improve the sustainability of fish farming industry.

## Summary and future perspectives

The use of immunonutritional approaches, such as novel feeds applied through nutritional programming, is gaining increasing interest. These approaches aim to coordinate immune-related mechanisms in fish (e.g., cellular and humoral responses in MALTs and lymphoid organs), modulate microbiota composition and function, and support fish growth. This can help control mortality, resistance to pathogens and increase fish welfare. Also, immunonutritional strategies could contribute to reducing economic losses in aquaculture associated with health problems during intensive production cycles. However, comparative research is still lacking to elucidate if the effects of bioactive ingredients or additives are species-specific or whether they depend on specific stages of fish development. In addition, efforts should be focused on strengthening the evaluation of overall fish performance and health under field conditions. This is crucial giving the emerging challenges related to climate change (e.g., more frequent pathogen outbreaks or emergence of new diseases), the raw material crisis (driven by e.g. climatic change, growing human population, as well as global health issues and political instabilities disturbing important supply chains) and growing public demand for more a more sustainable aquacultural production. Finally, one of the major bottlenecks remains the limited commercial availability of novel ingredients, largely due to low production volume and high cost, limited access to biomass for upscaling, and high processing costs.
